# Vitamin D and Forearm Fractures in Children Preliminary Findings: Risk Factors and Correlation between Low-Energy and High-Energy Fractures

**DOI:** 10.3390/children9050762

**Published:** 2022-05-23

**Authors:** Sinisa Ducic, Filip Milanovic, Mikan Lazovic, Bojan Bukva, Goran Djuricic, Vladimir Radlovic, Dejan Nikolic

**Affiliations:** 1 Pediatric Surgery Department, University Children’s Hospital, 11000 Belgrade, Serbia; sinisaducic@gmail.com (S.D.); mikanlazovic@gmail.com (M.L.); bojanbukva@yahoo.com (B.B.); vladimir.radlovic@udk.bg.ac.rs (V.R.); 2 Faculty of Medicine, University of Belgrade, 11000 Belgrade, Serbia; gorandjuricic@gmail.com (G.D.); denikol27@gmail.com (D.N.); 3 Radiology Department, University Children’s Hospital, 11000 Belgrade, Serbia; 4 Physical Medicine and Rehabilitation Department, University Children’s Hospital, 11000 Belgrade, Serbia

**Keywords:** forearm, fractures, bone metabolism, 25-hydroxyvitamin D, children

## Abstract

Background: The forearm is the most common fracture site in childhood, accounting for every fourth pediatric fracture. It is well described that vitamin D is involved in the regulation of bone mineralization and skeletal homeostasis by the regulation of calcium absorption. The aim of our study was to determine the influence of 25-hydroxyvitamin D levels on forearm fracture falls in a pediatric population, depending on level of energy impact. Additionally, we also aimed to evaluate the correlation between 25-hydroxyvitamin D levels and other tested risk factors for pediatric fractures. Methods: We evaluated 50 eligible children aged 3 to 12 years with a forearm fracture. According to energy impact, patients were grouped into low-energy fractures (LEF) and high-energy fractures (HEF) groups. The general characteristics of the patients included age, gender, sport participation, and fractured bone and its localization. We analyzed 25-hydroxyvitamin D, parathyroid hormone (PTH), calcium, magnesium, phosphate, C-reactive protein (CRP) levels, and body mass index (BMI). Results: There is a significant difference in the 25-hydroxyvitamin D levels distribution between LEF and HEF (*p* < 0.001) and PTH levels (*p* = 0.002). For magnesium levels, calcium levels, phosphate levels, and CRP levels, there were no significant differences in their frequency distribution. For the group of patients with LEF, there is a significantly positive correlation between 25-hydroxyvitamin D and calcium levels (*p* = 0.019) and a borderline significantly positive correlation between 25-hydroxyvitamin D and magnesium levels (*p* = 0.050). For the group of patients with HEF, there was only a significantly positive correlation between 25-hydroxyvitamin D and PTH levels (*p* < 0.001). Conclusions: Children with LEF were more frequently insufficient in 25-hydroxyvitamin D levels but had normal calcium levels, compared to the ones with HEF. These findings suggest that LEF and HEF in children might to a certain degree have different pathophysiological mechanisms.

## 1. Introduction

Bone fractures are common injuries in children, and it has been estimated that every second boy and almost every second girl (40%) have at least one fracture by 18 years of age [1]. A similar observation was presented in the study of Clark, where otherwise healthy boys aged 0–16 years have a lifetime risk for fracture between 42 and 64%, and girls between 27 and 40% [2]. Furthermore, the majority of fractures are on the upper limb in children [3]. The forearm is the most common fracture site in childhood, accounting for 25% of all pediatric fractures [1]. So far, the described risk factors for childhood fractures are obesity, lack of exposure to sunlight, lack of physical activity, darker skin pigmentation, milk avoidance, use of medications that lead to bone thinning, lower bone mass, and previous history of fracture [4,5,6,7,8]. It is well described that the vitamin D is involved in the regulation of bone mineralization and skeletal homeostasis by the regulation of calcium absorption. After being synthesized in the skin under the influence of ultraviolet light of the sun and further being activated in the processes of double hydroxylation in the liver and kidney, active metabolite enters the cell, binding to its specific receptor and subsequently to a responsive gene, such as that of calcium-binding protein, that mediates calcium absorption from the gut. The production of vitamin D is stimulated by parathyroid hormone (PTH) and decreased by calcium [5,6,7,8]. In some studies, it was shown that severe vitamin D deficiency causes rickets or osteomalacia, where the osteoid is not mineralized. On the other side, less severe vitamin D deficiency causes an increase in the serum PTH, leading to bone resorption, osteoporosis, and fractures [9]. Some authors have shown that the lower bone mineral density is associated with forearm fractures in adults and Caucasian children, while that risk has still not been investigated in African American children [10].

The importance of forearm fractures particularly of the distal radius refers to the fact that they can be used in the prediction of vertebral and hip fractures since they appear on average 15 years earlier than the hip fractures [11]. Moreover, Oyen et al. stated that vitamin D could influence other strength factors of the bone than the bone mineral density (BMD), since its levels, independently of the BMD differences, predicted the fractures [1]. As it was stated in the study of Hosseinzadeh et al., there are conflicting findings regarding the relationship of the low vitamin D levels and the risk of fractures [1].

The aim of our study was to determine the influence of 25-hydroxyvitamin D levels on forearm fracture falls in a pediatric population, depending on level of energy impact. Additionally, we also aimed to evaluate the correlation between the 25-hydroxyvitamin D levels and other tested risk factors in pediatric fractures.

## 2. Methods

In this prospective study, we evaluated 50 eligible children aged 3 to 12 years that were referred to the University Children’s Hospital for orthopedic treatment after a forearm fracture. The eligible participants were evaluated between November 2021 and March 2022. Prior to inclusion in the study, parents or legal guardians were informed about the study protocol and informed consent was obtained. The study followed the principles of good clinical practice and the Declaration of Helsinki and was approved by the Institutional Review Board of University Children’s Hospital, Belgrade, Serbia (approval number: 675/1, date: 1 April 2022).

The inclusion criteria for the study were as follows: present and diagnosed forearm fracture and positive anamnesis or heteroanamnesis for the fall prior to the fracture on the affected arm. 

Excluding criteria were the presence of chronic diseases and syndromes, negative history of previous fractures on the affected arm, acute infection or inflammation of the forearm bones, and patients that were on medications that might alter the vitamin D and calcium levels.

According to the energy impact, patients were grouped into low-energy fractures and high-energy fractures groups. Low-energy fractures occur as a result of falling from standing height or less, while high-energy fractures occur as a result of any other type of trauma (e.g., falling from height higher than standing height and motor vehicle accident) [12]. The fractures were diagnosed by radiographic imaging of the affected arm from the anterioposterior (AP) and lateral positions. 

The general characteristics of the patients included age, gender, sport participation, weekly milk intake, fractured bone and its localization, and performed surgery.

We further analyzed some other parameters: serum 25-hydroxyvitamin D, PTH, calcium, magnesium, phosphate, CRP levels, as well as BMI. 25-hydroxyvitamin D was measured from the serum taken from the study participants and analyzed by the ECLIA method from Roche Diagnostics. 25-hydroxyvitamin D levels were defined as sufficient (≥30 ng/mL), insufficient (10–29 ng/mL), and deficient (<10 ng/mL) [13]. Normal PTH, calcium, magnesium, phosphate, and CRP levels—and its deviations—were defined according to age [14,15]. According to the Centers for Disease Control and Prevention, BMI levels were calculated with regards to the age as underweight, healthy weight, overweight, and obese [16]. 

### Statistical Analysis

Results are presented as the mean value (MV) and standard deviation (SD) for continuous variables while for categorical variables as the whole numbers (N) and percentage (%). To evaluate the statistical difference of the categorical variables between the tested groups (low-energy and high-energy fractures), we used Chi-square and Fisher’s tests. For more than two comparisons, we introduced the Bonferroni correction to adjust the *p*-values. Spearman’s Rho correlation was used to access the degree and type of correlation between the vitamin D levels and other tested variables’ levels. Data were statistically analyzed using IBM SPSS software, version 21 (IBM Corporation, Armonk, NY, USA). Statistical significance was set at *p* < 0.05. 

## 3. Results

There were 32 (64%) patients with low-energy fractures and 18 (36%) patients with high-energy fractures. In both groups, male gender was more frequent (low-energy fractures—59.4%; high-energy fractures—66.7%) as well as those who participated in sports activities (low-energy—62.5%; high-energy fractures—72.2%). For those with low-energy fractures, almost four out of five patients (78.1%) had a fracture of both the radius and ulna; more than half of the patients in this group had a fracture of the distal part of the bone (59.4%) and 93.8% underwent surgery. Those with the high-energy fractures had a higher frequency of distal fractures (50%), while less than half of this sample had bone shaft fracture (44.4%). Fracture of the distal part of the bone was also more frequent in this group (every second patient, 50%). The majority of patients with LEF (93.8%) or HEF (83.3%) did not have surgery (Table 1).

There is a significant difference in the 25-hydroxyvitamin D levels distribution between low-energy and high-energy fractures (*p* < 0.001) and PTH levels (*p* = 0.002) (Table 2). For calcium levels, magnesium levels, phosphate levels, and CRP levels, there were no significant differences in their frequency distribution (Table 2).

For the group of patients with low-energy fractures, there is a significantly positive correlation between 25-hydroxyvitamin D and calcium levels (r = 0.412; *p* = 0.019) and a borderline significantly positive correlation between 25-hydroxyvitamin D and magnesium levels (r = 0.350; *p* = 0.050) (Table 3). For the group of patients with high-energy fractures, there was only a significantly positive correlation between 25-hydroxyvitamin D and PTH levels (r = 0.879; *p* < 0.001) (Table 3).

## 4. Discussion

Our results showed that the male predominance was higher for high-energy fractures, where two out of three were boys, while such an increased frequency of male gender of around 1.5 times was lower for low-energy fractures compared to the female gender. Similar findings were previously reported by different studies [17,18,19]. Such findings could suggest that there are differences in behavioral patterns between genders, particularly in terms of physical activity, where boys tend to be more active, especially in school playgrounds, and with aggressive behavior in certain conditions [20,21,22]. In the study of Lee et al., it was stated that the prevalence of physical aggression tends to decrease with age for girls, but not for boys [23]. Even though children worldwide are not meeting the WHO physical activity guidelines, our study sample has shown that less than two-thirds of those with low-energy fractures and less than three out of four children with high-energy fractures were engaged in sports activities [24,25]. From the WHO report in 2020, it was recommended that children and adolescents from 5 to 17 years of age should engage in 60 min of moderate to vigorous intensity physical activity on average per day, which should be mostly aerobic [24]. Furthermore, in the systematic review of Donnelly et al., the authors stated the importance of physical activity on cognitive function in children [26]. In support of these findings, another systematic review of Bidzan-Bluma and Lipowska has found that there are positive benefits to cognitive and emotional functions, even after engaging in sport activities of children in later childhood [27]. Finally, in the Centers for Disease Control and Prevention (CDC) report, it was stated that 10−50% of bone mass and structure are due to diet and physical activity [28]. Regular physical activity along with vitamin D increases positive calcium effects [28].

Furthermore, we have demonstrated that just below two thirds of low-energy fractures were in tested subjects with insufficient vitamin D levels, while for those with acquired high-energy fractures, just above two out of five were with deficient vitamin D levels. In the study of Thompson et al., it was found that 65% of pediatric patients with fractures had a deficiency in or insufficient vitamin D levels [29]. Moreover, in the systematic review and meta-analysis of Yang et al., it was noticed that pediatric patients with fractures had lower levels of serum 25(OH) vitamin D [30]. Additionally, Moore et al. have shown that 38% of children with low-energy fractures were deficient in 25-hydroxyvitamin D [31]. Such findings are to a certain degree in correlation with our results.

The possible discrepancies between low- and high-energy fractures in our study regarding the frequencies of 25-hydroxyvitamin D level deficiency might be explained to a certain degree by the assumption that the low-energy fractures are likely to be associated with an impaired bone quality [30]. Thus, this postulation could point the fact that in the group of low-energy fractures, most of the tested subjects had insufficient rather that deficient levels of 25-hydroxyvitamin D compared to the group with a high-energy fracture.

Additionally, our study has demonstrated that for the group with LEF, the PTH levels were abnormal in around every fifth patient. These findings point to the assumption that PTH levels does not necessarily reflect the 25-hydroxyvitamin D level changes for the group of LEF. Moreover, every second patient has lower PTH levels in the group of HEF. It is worth noticing that despite the contradictory findings in our study, there is a positive significant correlation between PTH levels and 25-hydroxyvitamin D levels. These results have shown that, for the HEF group, being deficient in 25-hydroxyvitamin D along with lower PTH levels could be somehow a predisposing factor for such a type of fracture.

Our findings pointed out that just above every second patient with LEF had normal Ca levels while two out of five had lower levels. Additionally, it was shown that there is a positive significant association between the Ca and 25-hydroxyvitamin D levels. In the study of El-Sakka et al., the significant correlation between vitamin D levels and serum calcium levels was found in a pediatric population with forearm fractures [32]. Moreover, it should be worth mentioning that the skeletal fragility predictors in adults are inadequate calcium intake as well as distal forearm fractures in a pediatric population [32]. Therefore, proper calcium monitoring and management along with prevention of the distal forearm fractures in childhood should be advised in clinical practice, with adequate education of parents or legal guardians for children at risk. Additionally, in the study of Maatta et al., gender was shown to have an influence on low-energy fractures, where in boys, aside from bone strength, other factors were shown to increase the risk for fracture, including poor balance, excess body fat, and low physical activity [33].

Additionally, for HEF group, we have demonstrated that almost every third patient had normal or lower Ca levels. Our results for the Ca levels in the HEF group does not follow the changes in PTH levels, pointing out that the patients with HEF could have other predisposing factors for the fracture.

There are several limitations to this study. The first one refers to the number of participants. The lower number might affect the sensitivity of the obtained results. An additional limitation includes the age group: older children might have other predisposing factors for fractures that could affect the mechanism behind a low- or high-energy fracture pattern. Furthermore, in this study, there is a lack of assessment in different seasons (proportionally spring, summer, autumn and winter), as well as a lack of use of the Cole index. Thus, other studies are needed.

Since we have demonstrated the presence of differences in certain parameters between LEF and HEF, future studies, preferably on a larger sample of patients, should evaluate the potential predictors of the evaluated parameters on the forearm fracture injury mechanism in children. Furthermore, the potential role of comorbidities should be considered in future evaluations of their influence on the forearm fracture mechanism.

## 5. Conclusions

Our results provide additional knowledge on the role of vitamin D and calcium levels in forearm fractures in children. We have demonstrated that children with low-energy fractures were more frequently insufficient in 25-hydroxyvitamin D levels, but had normal calcium levels, compared to ones with high-energy fractures. These findings suggest that low- or high-energy forearm fractures in children might, to a certain degree, have different pathophysiological mechanisms. Therefore, a personalized approach, as well as implementation of preventive measures, such as vitamin D supplementation and calcium levels monitoring, should result in a reduction in these types of fractures.

## Figures and Tables

**Table 1:** General characteristics of the tested sample.

Variables		Low-Energy Fractures N = 32	High-Energy Fractures N = 18	Total N = 50
Age (years) (MV ± SD)		8.03 ± 2.15	9.06 ± 1.98	8.40 ± 2.13
Weekly milk intake (mL) (MV ± SD)		1578.13 ± 265.17	1972.22 ± 269.65	1720.00 ± 325.92
Gender N (%)	Male	19 (59.4%)	12 (66.7%)	31 (62%)
	Female	13 (40.6%)	6 (33.3%)	19 (38%)
Sport N (%)	Yes	20 (62.5%)	13 (72.2%)	33 (66%)
	No	12 (37.5%)	7 (38.9%)	19 (38%)
Bone N (%)	Radius	5 (15.6%)	8 (44.4%)	13 (26%)
	Ulna	2 (6.3%)	1 (5.6%)	3 (6%)
	Radius and Ulna	25 (78.1%)	9 (50%)	34 (68%)
Fracture site N (%)	Bone Shaft	11 (34.4%)	5 (27.8%)	16 (32%)
	Proximal Part	2 (6.3%)	4 (22.2%)	6 (12%)
	Distal Part	19 (59.4%)	9 (50%)	28 (56%)
Surgery N (%)	No	30 (93.8%)	15 (83.3%)	45 (90%)
	Yes	2 (6.2%)	3 (16.7%)	5 (10%)

**Table 2:** Frequencies of the tested variables with regards to low-energy and high-energy fractures.

Parameters		Low-Energy Fractures N = 32 N (%)	High-Energy Fractures N = 18 N (%)	*p*-Value
25-hydroxyvitamin D levels	Normal	11 (34.4)	6 (33.3)	<0.001 *
	Insufficiency	20 (62.5)	4 (22.2)	
	Deficiency	1 (3.1)	8 (44.4)	
PTH levels	Normal	25 (78.1)	7 (38.9)	0.002 *
	Hyper-	5 (15.6)	2 (11.1)	
	Hypo-	2 (6.3)	9 (50)	
Calcium levels	Normal	18 (56.3)	6 (33.3)	0.029 *
	Hyper-	1 (3.1)	5 (27.8)	
	Hypo-	13 (40.6)	7 (38.9)	
Magnesium levels	Normal	29	17	>0.05 **
	Hyper-	0	0	
	Hypo-	3	1	
Phosphate levels	Normal	26	15	>0.05 **
	Hyper-	0 (0%)	0 (0%)	
	Hypo-	6	3	
CRP levels	Normal	9	3	>0.05 **
	Increased	23	15	
BMI levels	Underweight	4	2	>0.05 *
	Normal Weight	16	10	
	Overweight	6	3	
	Obesity	6	3	

* Chi square test; ** Fisher test.

**Table 3:** Correlation between the 25-hydroxyvitamin D levels and tested variables.

25-Hydroxyvitamin D Levels	Low-Energy Fractures		High-Energy Fractures		Total	
	r *	*p*	r *	*p*	r *	*p*
PTH levels	−0.106	0.565	0.879	<0.001	0.495	<0.001
Calcium levels	0.412	0.019	0.247	0.322	−0.054	0.711
Magnesium levels	0.350	0.050	0.251	0.315	0.272	0.056
Phosphate levels	−0.169	0.355	0.463	0.053	0.094	0.515
CRP levels	−0.125	0.497	−0.123	0.626	−0.069	0.635
BMI levels	0.093	0.614	0.126	0.618	0.103	0.475

* Spearman’s Rho correlation.
